# Ex vivo glucocorticoid receptor-mediated IL-10 response predicts the course of depression severity

**DOI:** 10.1007/s00702-020-02288-7

**Published:** 2021-01-15

**Authors:** Claudia von Zimmermann, Lea Böhm, Tanja Richter-Schmidinger, Johannes Kornhuber, Bernd Lenz, Christiane Mühle

**Affiliations:** 1 Department of Psychiatry and Psychotherapy, Friedrich-Alexander University Erlangen-Nürnberg (FAU), Schwabachanlage 6, 91054 Erlangen, Germany; 2 Department of Addictive Behavior and Addiction Medicine, Central Institute of Mental Health (CIMH), Medical Faculty Mannheim, Heidelberg University, Mannheim, Germany

**Keywords:** Depression, Glucocorticoid receptor, Hypothalamic–pituitary–adrenal axis, Cytokines

## Abstract

Directly measuring hypothalamic pituitary adrenal (HPA) axis function, an important player in affective disorders, is intensive and invasive. A crucial component of this system, the activity of the glucocorticoid receptor (GR), can be assessed ex vivo instead. Here, we investigated GR sensitivity in patients with major depressive disorder (MDD) to determine its predictive potential. Psychometric data and blood samples were collected from patients experiencing a major depressive episode (MDE, *n* = 87), healthy control subjects (*n* = 49), and patients with remitted MDD (*n* = 31) at baseline and (for patients) after median 20 days of follow-up after treatment as usual. Blood cells were stimulated ex vivo with lipopolysaccharide and the effect was suppressed by increasing dexamethasone (DEX) concentrations. The resultant cytokine secretion profile (for IL-6, IL-10, and TNF-α) was considered indicative of GR activity. Higher baseline scores of the Montgomery–Åsberg Depression Rating Scale (MADRS) were associated with a stronger decrease of logIC IL-6 (indicating an increase of GR sensitivity). Higher baseline logEC IL-10 (indicating a lower GR sensitivity) and a stronger reduction of logEC IL-10 (indicating a stronger increase in GR sensitivity) were associated with a stronger decrease in the MADRS score. Patients with remitted MDD showed higher logIC TNF-α values (indicating lower GR sensitivity) in comparison to patients with a current MDD at baseline and follow-up. Initially low GR sensitivity measured ex vivo in peripheral blood cells that increases over the course of treatment could serve as a predictive marker for stronger improvement in depression severity.

## Introduction

Major depressive disorder (MDD) is an important public health issue and one of the five leading reasons that caused people to live with a disability in 2016 (GBD 2016 disease and injury incidence and prevalence collaborators 2017). In addition to the monoamine hypothesis, other mechanisms have been suggested to cause depression such as dysfunction of the hypothalamic–pituitary–adrenal (HPA) axis with pathological glucocorticoid balance (Belmaker and Agam 2008; Holsboer 2000) and altered neurogenesis potentially involving the ceramide system (Dinoff et al. 2017; Gulbins et al. 2015; Kornhuber et al. 2009; Mühle et al. 2019a, b; Rhein et al. 2017). Furthermore, inflammatory processes are associated with the symptoms of MDD (Kiecolt-Glaser et al. 2015; Wohleb et al. 2016). Stress exposure can induce proinflammatory cytokines including the transcription factors nuclear factor kappaB (Bierhaus et al. 2003) and interleukin 6 (IL-6) (Pace et al. 2006). It has been assumed that “sickness behavior” promotes host survival during infections (Raison and Miller 2013). The interaction of the immune system with neurocircuits seems to modulate the risk for depression (Haapakoski et al. 2016; Miller and Raison 2016). Elevated proinflammatory parameters are also associated with a worse response to antidepressants (Miller et al. 2009; Raison et al. 2013). Hence, the inflammatory process is a new target for therapeutic strategies like the administration of tumor necrosis factor-alpha (TNF-α) antagonists (Bekhbat et al. 2018; Raison et al. 2013) or anti-inflammatory antibiotics (Cai et al. 2020; Husain et al. 2017). In addition, treatment with antidepressant drugs can lead to alterations in peripheral cytokine levels (Köhler et al. 2017; Liu et al. 2020).

Glucocorticoids influence inflammatory processes in the brain (Nadeau and Rivest 2003) and dysregulation of the HPA axis plays a critical role in MDD (Carroll et al. 1968; Mokhtari et al. 2013). The combined dexamethasone–corticotropin-releasing hormone (DEX/CRH) test, a measure of HPA axis (dys)regulation, is altered in 24–35% of patients with acute depression (Schüle et al. 2009). Heuser et al. (1994) found a sensitivity of the DEX/CRH test for MDD of even up to 80%, depending on the age of the patients. Impaired glucocorticoid receptor (GR) function seems to be one reason for this dysregulation (Busch and Menke 2019; Mokhtari et al. 2013; Silverman and Sternberg 2012). Restoring GR function, an effect of antidepressant treatment, is one major aspect of treating depression (Anacker et al. 2011; Carvalho and Pariante 2008). Moreover, early improvement in HPA axis function is associated with successful antidepressive therapy (Ising et al. 2007). The combined DEX/CRH test requires several blood draws as well as systemic administration of DEX, both of which represent additional burdens to the patient. To date, pharmacological treatment options for this heterogeneous illness are still limited. Even though a subset of affected patients exhibits altered HPA axis function, no routine test is used clinically to identify this dysregulation nor is any specific treatment initiated to normalize HPA axis function. GR function, as a part of the HPA axis, could serve as a biomarker for detecting and distinguishing subforms of MDD and might help to develop more individualized therapeutic approaches. Given that not all patients with depression show alterations in the HPA axis (Menke 2019), there is a need for a less burdensome ex vivo assessment. Several methods to measure GR sensitivity have been described; these include the inhibition of peripheral blood mononuclear cell (PBMC) proliferation (Chriguer et al. 2005), the DEX-induced upregulation of glucocorticoid-responsive genes (e.g., *GILZ*, *FKBP51*), and the DEX-induced repression of lipopolysaccharide (LPS)-induced cytokines (e.g., IL-6, TNF-α) (Bellingrath et al. 2013; Burnsides et al. 2012). TNF-α seems to be sensitive to suppression by DEX, at least in patients with chronic fatigue syndrome (Lynn et al. 2018). This could be due to stronger inhibition of Th1 over Th2 CD4 + T cells through glucocorticoids (Lynn et al. 2018; Visser et al. 2000). In addition to this, LPS can also modulate the production of the anti-inflammatory cytokine IL-10 (Saraiva and O’Garra 2010; van den Bosch et al. 2014) and measuring LPS-induced IL-10 response has been shown to be a sensitive marker for disturbed glucocorticoid regulation at least in patients with chronic fatigue syndrome (Visser et al. 2001). In this study, we investigated an easily applicable, less burdensome ex vivo stimulation method to measure GR sensitivity alterations in patients with depression based on published protocols (Bellingrath et al. 2013; Burnsides et al. 2012; Smits et al. 1998; ter Wolbeek et al. 2008). Therefore, whole blood was stimulated with LPS and the production of the cytokines IL-6, IL-10, and TNF-α as well as suppression of this effect by increasing concentrations of DEX has been measured.

### Aims of the study

We hypothesized that GR sensitivity relates to the severity and course of depression. In this study, we aimed to measure GR sensitivity indirectly at baseline and after receiving standard care for median 20 days. Therefore, we used an ex vivo assessment of the production of the cytokines IL-6, IL-10, and TNF-α by leukocytes after stimulation of whole blood with LPS and inhibition of this effect by increasing concentrations of DEX.

## Methods

### Sample population

We analyzed data collected for the CeraBiDe (“Ceramide-associated Biomarkers in Depression”) study (Mühle et al. 2019b; von Zimmermann et al. 2020; Wagner et al. 2019), which was approved by the Ethics Committee of the Medical Faculty of the Friedrich-Alexander University Erlangen-Nürnberg (FAU, ID 148_13 B, 2013).

We recruited patients with a current MDE, healthy control subjects, and patient with a remitted MDD between 01/2014 and 01/2017. All participants provided their written informed consent. The patients were recruited from the in- and outpatients of the Department of Psychiatry and Psychotherapy at the University Hospital Erlangen, in addition to further interested people fulfilling the inclusion criteria, informed about the study via letters, local newspapers, flyers, and via Internet advertisement. Healthy control subjects were local citizens.

All participants underwent a multi-step screening procedure to exclude severe physical (e.g., autoimmune disorder, cancer) and psychiatric morbidities (with the exclusion of comorbid anxiety disorder and nicotine dependence, for healthy control subjects’ exclusion of all psychiatric morbidities except for nicotine dependency), the use of corticosteroids or anti-inflammatory drugs in the past 7 days, pregnancy, and breastfeeding. We included 87 patients currently undergoing an MDE, 49 healthy control subjects, and 31 patients with remitted MDD. Participants ranged in age from 19 to 74 years and in body mass index (BMI) from 18.7 to 34.9 kg/m^2^. For diagnosis and exclusion of psychiatric comorbidities, we used the structured clinical interview from the DSM-IV (SKID-I) and quantified depression severity using the Beck Depression Inventory (BDI)-II, the 17-item Hamilton Depression Rating Scale (HAMD), and the Montgomery–Åsberg Depression Rating Scale (MADRS). 84 of the 87 patients with a current MDE participated in a direct follow-up (14–43 days post inclusion, median 20 days, interquartile range [IQR] 15–27). All patients received treatment as usual during the follow-up period.

### Blood analysis

Blood samples were collected in the morning after an overnight fast. The leukocytes were quantified at the Institute of Transfusion Medicine of the University Hospital Erlangen (D-ML-13297-01 accredited).

### Ex vivo stimulation

Stimulation with LPS induces higher production of IL-6 and TNF-α and inhibits production of IL-10 in leukocytes in vitro. Addition of DEX inhibits these effects. Based on published protocols for ex vivo GR sensitivity assessment (Bellingrath et al. 2013; Burnsides et al. 2012; Smits et al. 1998; ter Wolbeek et al. 2008), modified conditions with optimized incubation time and LPS and DEX concentrations specifically for this application were used in this study. For the stimulation trial, increasing amounts of DEX (D1756, Sigma-Aldrich, Darmstadt, Germany) at final concentrations of 0 nM, 0.01 nM, 0.10 nM, 0.32 nM, 1.00 nM, 3.2 nM, 10 nM, 32 nM, 100 nM, 316 nM, 1.00 µM, 3.16 µM, 10 µM, and 100 µM in PBS were combined with 250 ng/ml LPS (L4931, Sigma-Aldrich, final concentration) in PBS each and stored alongside a duplicate of PBS alone in PCR strips as a total of 16 single-use aliquots of 20 µl at − 20 °C. Within 1 h of collection, 230 µl of lithium–heparin-treated whole blood was added to each thawed, pre-prepared 20 µl aliquot of LPS/DEX (reaching the given final concentrations), in duplicate, mixed thoroughly but gently, and incubated for exactly 5.0 h at 37 °C in an incubator. Subsequently, the samples in PCR strips were centrifuged for 5 min at 2000 g and 100 µl of the plasma supernatant was collected and stored at − 80 °C.

### Quantification of IL-6, IL-10, and TNF-α

Plasma levels of TNF-α were assayed using the sandwich Human TNFalpha DuoSet ELISA (6 µl sample, standard range 600–6 pg/ml, intra-assay coefficient of variation [cv] of 2%, inter-assay cv of 23%, DY210, R&D Systems, Bio-Techne GmbH, Wiesbaden, Germany). Plasma levels of IL-6 were quantified by the sandwich Human IL-6 DuoSet ELISA (3 µl sample, standard range 1500–9 pg/ml, intra-assay cv of 1%, inter-assay cv of 7%, DY206, R&D Systems, Bio-Techne GmbH). Plasma levels of IL-10 were determined with the sandwich Human IL-10 DuoSet ELISA (100 µl sample, standard range 1000–4 pg/ml, intra-assay cv of 6%, inter-assay cv of 25%, DY217, R&D Systems, Bio-Techne GmbH). All of the samples from one stimulation were always run on the same plate.

### Statistical analyses

We calculated the logIC50 IL-6, logEC50 IL-10, and logIC50 TNF-α values, i.e., the log10DEX concentrations needed to suppress 50% of the LPS-induced or -inhibited IL-6, IL-10, and TNF-α secretion. SPSS for Windows 24.0 (SPSS Inc., Chicago, IL, USA) and GraphPad Prism 5 (GraphPad Software Inc., San Diego, CA, USA) were used to analyze the data. Since the data were not normally distributed, non-parametric methods were used and medians and interquartile ranges (IQR), calculated using the custom tables function, are reported for continuous data. We employed the Mann–Whitney *U* test to compare independent groups, the Wilcoxon test for longitudinal differences in dependent groups, and Spearman’s method for bivariate correlations. The Chi-square test was used to test for differences in frequencies. *P* < 0.05 was considered statistically significant. We were able to determine complete logIC/EC values in 167 study subjects at baseline and 79 subjects at follow-up.

## Results

### Cohort characteristics

The patients with a current MDE did not significantly differ from the healthy control subjects by sex, relationship status, age, or education level (years), but were significantly more likely to be divorced and scored significantly higher on the BDI-II, the HAMD, and the MADRS.

The patients with a current MDE also did not differ from those with remitted MDD in terms of relationship status, age, or education level (years); however, they were significantly more likely to be male and scored significantly higher on the BDI-II, the HAMD, and the MADRS (Table 1).

**Table 1:** Cohort characteristics and group differences

	Patients with a current MDE (*N* = 87)				Healthy control subjects (*N* = 49)				Patients with a remitted MDD (*N* = 31)				Patients with a current MDE vs. healthy control subjects		Patients with a current MDE vs. patients with a remitted MDD	
	*N*	*F*/median IQR			*N*	*F*/median IQR			*N*	*F*/median IQR			*χ*^2^, *df*/*U*	*P*	*χ*^2^, *df*/*U*	*P*
Baseline																
Females (%)	87	54.0			49	55.1			31	77.4			< 0.1, 1	0.903^a^	5.2, 1	**0.022**^a^
Single (%)	87	35.6			49	26.5			31	32.3			1.2, 1	0.276^a^	0.1, 1	0.735^a^
Married (%)	87	42.5			49	38.8			31	58.1			0.2, 1	0.669^a^	2.2, 1	0.137^a^
Divorced (%)	87	20.7			48	6.3			30	36.7			4.9, 1	**0.027**^a^	3.1, 1	0.081^a^
Age (years)	87	46	34	54	49	43	33	56	31	49	46	58	2128	0.987^b^	1051	0.069^b^
Sum of education years	79	15	13	17	40	15	13	17	27	14	13	16	1548	0.854^b^	990	0.576^b^
BDI-II	87	28	21	34	49	1	0	3	31	3	0	5	3	**< 0.001**^**b**^	58	**< 0.001**^**b**^
HAMD	87	22	19	26	49	1	0	2	31	1	0	3	0	**< 0.001**^**b**^	0	**< 0.001**^**b**^
MADRS	87	27	23	32	49	0	0	1	31	1	0	4	0	**< 0.001**^**b**^	0	**< 0.001**^**b**^
Leukocytes (10^3^/µl)	87	5.69	4.90	6.94	49	5.17	4.58	6.13	31	6.21	5.00	7.04	1707	0.054^b^	1218	0.423^b^
Follow-up																
BDI-II	84	19	14	28												
HAMD	84	18	12	21												
MADRS	84	20	15	26												
Leukocytes	84	5.69	4.95	7.19												

The table reports relative frequencies (*F*) and medians (IQR)

*P* < 0.05 in bold print

*MDE* Major depressive episode, *MDD* Major depressive disorder, *IQR* interquartile range, *BDI-II* Beck Depression Inventory II, *HAMD* Hamilton Depression Rating Scale, *MADRS* Montgomery and Åsberg Depression Rating Scale

^a^*χ*^2^ test

^b^Mann–Whitney *U* test

### Group differences and time course

The patients with a current MDE did not significantly differ from the healthy control subjects in terms of cytokine response and from patients with remitted MDD in terms of logIC IL-6 and logEC IL-10, both at baseline and the follow-up.

However, we found significantly higher logIC TNF-α in patients with remitted MDD than in patients with a current MDE at both time points (Table 2).

**Table 2:** Group differences for logEC and logIC values for cytokines

	*N*	Median	IQR		Versus patients with a current MDE			
					At baseline		At follow-up	
Patients with a current MDE at baseline								
IL-6	87	1.47	1.33	1.60				
IL-10	87	1.60	1.40	1.84				
TNF-α	87	1.35	1.25	1.50				
Patients with a current MDE at follow-up					*z*	*P*^a^		
IL-6	79	1.52	1.36	1.62	− 1.0	0.325		
IL-10	79	1.68	1.43	1.80	− 1.2	0.237		
TNF-α	79	1.36	1.27	1.50	− 0.4	0.724		
Healthy control subjects					*U*	*P*^b^	*U*	*P*^b^
IL-6	49	1.49	1.32	1.68	1979	0.489	1932	0.984
IL-10	49	1.66	1.43	1.87	1992	0.527	1840	0.638
TNF-α	49	1.35	1.27	1.46	2112	0.928	1901	0.866
Patients with a remitted MDD					*U*	*P*^b^	*U*	*P*^b^
IL-6	31	1.55	1.39	1.66	1056	0.074	1083	0.345
IL-10	31	1.75	1.52	1.85	1153	0.232	1081	0.339
TNF-α	31	1.47	1.30	1.60	1014	**0.041**	919	**0.042**

The table reports median and IQR of logIC values for IL-6 and TNF-α and logEC values for IL-10

*P* < 0.05 in bold print

*MDE* major depressive episode, *MDD* major depressive disorder, *IQR* interquartile range

^a^Wilcoxon signed-rank

^b^Mann–Whitney *U* test

LogIC IL-6, logEC IL-10, and logIC TNF-α did not significantly change between baseline and the follow-up in patients currently undergoing an MDE.

### Correlations in patients with a current MDE

**Fig. 1:**
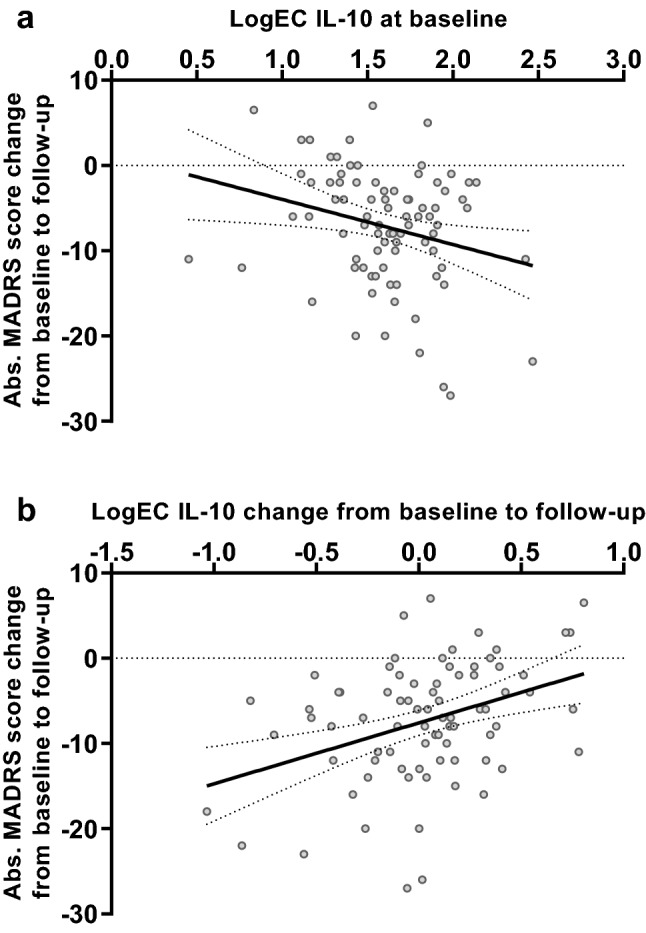
The change in absolute MADRS scores from baseline to follow-up was significantly negatively correlated with logEC IL-10 at baseline (**a**) and significantly positively with the change in logEC IL-10 (**b**). The graphs contain best-fit lines from regression analyses with 95% confidence intervals

Neither the logIC IL-6, logEC IL-10, and logIC TNF-α values at baseline, follow-up, nor their overall courses significantly correlated with depression severity scores or course measured by the BDI-II or the HAMD (data not shown). However, the course of MADRS scores correlated significantly negatively with logEC IL-10 at baseline and positively with logEC IL-10 course (Fig. 1); moreover, baseline MADRS scores significantly negatively correlated with the logIC IL-6 course (Table 3). There were no other significant correlations with MADRS scores or course.

**Table 3:** Spearman correlations between logEC and logIC values for cytokines and MADRS scores for depression severity

	Baseline			Follow-up			Course (absolute change)		
	IL-6	IL-10	TNF-α	IL-6	IL-10	TNF-α	IL-6	IL-10	TNF-α
MADRS									
Baseline									
*N*	87	87	87	79	79	79	79	79	79
*ρ*	0.037	0.095	0.077	− 0.164	− 0.116	− 0.044	− 0.226	− 0.188	− 0.078
*P*	0.732	0.381	0.478	0.148	0.308	0.699	**0.046**	0.096	0.493
Follow-up									
*N*	84	84	84	79	79	79	79	79	79
*ρ*	− 0.068	− 0.132	0.036	0.018	− 0.037	0.044	0.028	0.169	− 0.031
*P*	0.541	0.231	0.742	0.872	0.746	0.700	0.805	0.136	0.789
Course (absolute change)									
*N*	84	84	84	79	79	79	79	79	79
*ρ*	− 0.062	− 0.230	− 0.050	0.140	0.091	0.079	0.160	0.333	0.024
*P*	0.577	**0.036**	0.653	0.219	0.427	0.492	0.159	**0.003**	0.836

*P* < 0.05 in bold print

*MADRS* Montgomery and Åsberg Depression Rating Scale

## Discussion

In this study, we analyzed the relationship between depression, depression severity, the course of depression, and GR sensitivity in an ex vivo stimulation trial. We determined the logIC50 or logEC50 values for IL-6, IL-10, and TNF-α after stimulation with LPS and dose-dependent suppression with DEX.

This is the first study to show that, in patients with depression, a decrease in depression severity from baseline to follow-up, measured by the MADRS, was associated with lower GR sensitivity (indicated by higher logEC IL-10) at baseline and an increase in GR sensitivity (indicated by a reduction of logEC IL-10) from baseline to follow-up. Thus, we observed an initially lower GR sensitivity and its shift toward higher GR sensitivity in patients with greater improvement in their depressive symptoms. In line with our results, an association between remission and significantly higher cortisol response in the DEX/CRH test at baseline has been shown in male but not in female patients with depression (Binder et al. 2009). Our results are, in part, in accordance with Schüle et al. (2009) and Ising et al. (2007), who found that improved HPA responsiveness within the first 1–3 weeks of treatment predicts improvement of depressive symptoms. Furthermore, complete normalization of the HPA axis seems to be associated with recovery from depression (Behnken et al. 2013; Binder et al. 2009; Hardeveld et al. 2014; Holsboer et al. 1982; Kunugi et al. 2006; Rybakowski and Twardowska 1999; Tanke et al. 2008).

Also, an improvement in GR function (Reppermund et al. 2007) or a partial recovery (Lisi et al. 2013) during treatment has been shown before. Contrary to our findings, non-suppression in the DEX/CRH test at baseline was not related to later therapeutic response in a former study (Schule et al. 2009), which could be explained by different study populations. Moreover, the response to a baseline DEX suppression test is not predictive of short-term response, according to a meta-analysis (Ribeiro et al. 1993).

Contrary to our expectations and despite the notion that impaired GR signaling is a key element underlying the development of depression (Anacker et al. 2011; Holsboer 2000), we did not find any difference in the GR sensitivity (i.e., DEX-induced changes in IL-10, IL-6, and TNF-α) of patients with depression compared with healthy control subjects at baseline. Miller et al. (2005) also did not find differences in the IC50 of IL-6 and TNF-α. In contrast to other studies (Meador-Woodruff et al. 1987), we found no association between initial GR function and depression severity at baseline.

High baseline depression severity (MADRS scores) predicted an increase of GR sensitivity (assessed via a stronger decrease of logIC IL-6) during the follow-up period which agrees with the observations that a stronger decrease in MADRS score (improvement of depression severity) during the follow-up correlated with a stronger increase in GR sensitivity (assessed via a stronger reduction of logEC IL-10) during the follow-up and that a stronger decrease in MADRS score was predicted by lower baseline GR sensitivity (assessed via higher logEC IL-10). IL-10 assessment might be more sensitive to changes in depression severity. These findings require replication as most prior research on GR sensitivity determined ex vivo has focused on cytokines other than IL-10.

Our specific study population may also explain some of the unexpected observations. We did not further differentiate our patients according to MDD subtype; for example, anxious depression, a subtype of MDD associated with increased GR sensitivity (Menke et al. 2018). We also excluded patients with suicidal tendencies, as HPA axis hyperactivity and decreased GR sensitivity are characteristics of suicidal patients (Coryell and Schlesser 2001; Jokinen et al. 2007; Jokinen and Nordström 2009; Lenz et al. 2019). Moreover, patients with chronic depression do not differ from healthy control subjects on the DEX suppression test (Watson et al. 2002). We also did not subdivide our cohort according to whether their depressive episode was singular or recurrent, which may further explain the missing effect in the present study. For the complete group of patients with depression, we did not find any change between baseline and follow-up in GR sensitivity as reflected by logIC IL-6, logEC IL-10, and logIC TNF-α. This could be due to the heterogeneity of our patient sample.

Patients with remitted MDD, who had not suffered from depression for at least 12 months, had lower GR sensitivity (measured by logIC TNF-α) than patients currently undergoing an MDE. We cannot provide follow-up data, information on previous depressive episodes, or HPA dysregulation during their acute depressive episode, which might be interesting in comparison to the currently depressed patients. Patients who experienced depression and remission have not been widely studied. Further examination is warranted, particularly because depression is a chronic illness, and with HPA axis disturbance preceding relapse (Appelhof et al. 2006; Aubry et al. 2007; Holsboer et al. 1982; Zobel et al. 2001). Moreover, studies also have shown clinical remission despite persisting GR dysfunction (Schule et al. 2009).

We found changes in depression severity from baseline to follow-up were associated with GR sensitivity when measured by the MADRS, but no significant effect with the BDI-II or HAMD. This could be caused by the different sensitivity of the rating scales to changes in depression severity (Montgomery and  Åsberg 1979) or the different sensitivity of the rating scales to different subtypes of depressive illness. While the HAMD and MADRS are both based on a clinical interview with the patients, the scales differ in part in terms of the symptoms assessed, which could also account for the different results. The lack of significance when using the BDI-II or HAMD could also be due to the small sample size.

Sex differences in depression are well known (Rubinow and Schmidt 2019). The limited number of participants did not allow us to study men and women separately despite these groups exhibiting potentially different effects that could mask correlations in the total group.

We conducted many statistical tests; thus, we cannot rule out that some of our findings may represent false positives. Validation in future studies is certainly needed. Although, as above-mentioned, numerous studies have shown restored GR function upon recovery from depression, other reports describe a persistence of GR dysfunction after remission (Pintor et al. 2007), some particularly in patients with bipolar disorder (Hennings et al. 2009; Rybakowski and Twardowska 1999).

Our study has further limitations. We used an associational study design, which does not allow causal conclusions to be drawn. We analyzed a rather small study population and excluded many participants from the whole sample (Wagner et al. 2019) due to data quality control. We did not differentiate according to clinical characteristics such as episode duration or former episodes. Possible confounding factors [e.g., chronic fatigue syndrome (Tomas et al. 2013), childhood trauma (Lynn et al. 2018)] could have influenced our results.

To reduce this bias we excluded other psychiatric comorbidities like post-traumatic stress disorder, which is a strength of our study. Furthermore, we excluded any patients taking anti-inflammatory medications. The ex vivo stimulation trial design was deemed less burdensome for the patients than the common DEX/CRH test.

In summary, our results support the importance of the GR in the pathology and progression of depression and provide a foundation for further analyses.

## Data Availability

Data are available upon request.
